# The Value of Crop Production and Pollination Services in the Eastern Amazon

**DOI:** 10.1007/s13744-020-00791-w

**Published:** 2020-06-15

**Authors:** R C Borges, R M Brito, V L Imperatriz-Fonseca, T C Giannini

**Affiliations:** 1 Instituto Tecnológico Vale Desenvolvimento Sustentável, Rua Boaventura da Silva, 955, Nazaré, Belém, Pará 66055-090 Brasil; 2 Univ. Federal do Pará, Belém, Pará Brasil; 3 Univ. de São Paulo, São Paulo, Brasil

**Keywords:** Nature’s contribution to people, food security, human well-being, economic vulnerability, açaí, global changes

## Abstract

Nature safeguards living organisms and the ecosystem functions and services delivered by them. Animal pollination is an important Ecosystem Service since it plays a key role for achieving the sustainable development goals by safeguarding worldwide food production. Thus, conservation of pollination services is a major priority for guaranteeing global food security in the long term. Here we evaluate the crop pollination services in Pará state (Eastern Amazon, Brazil) focusing on two questions: (1) What is the economic value of crop production and pollination service in Pará? (2) Which municipalities are most dependent on pollination services considering local economies? We found 36 crops produced in the state; 20 (55%) crops are dependent on animal pollinators. In 2016, crop production value (CPV) for Pará state was US$ 2.95 billion and total pollination service value (PSV) was US$ 983.2 million, corresponding to 33% of CPV in Pará. Highest PSV value crops were açaí palm (US$635.6 million), cocoa (US$187.6 million), soybean (US$98.4 million), and watermelon (US$26.1 million), accounting for 96% of Pará’s PSV. Two municipalities (Medicilândia and Igarapé Miri) presented more than 50% of their GDP based on pollination services. In general, we found low crop diversity in the municipalities of Pará, suggesting an economic rural vulnerability for the state, mainly supported by the high productions of soy and açaí. Pollinator conservation and ecological intensified farming practices are urgent for supporting sustainable development for the state.

**Electronic supplementary material:**

The online version of this article (10.1007/s13744-020-00791-w) contains supplementary material, which is available to authorized users.

## Introduction

Nature safeguards living organisms and the ecosystem functions and services delivered by them; however, the ongoing anthropogenic-induced global changes resulted in an unprecedented decline in biodiversity and its contributions to people (Diaz *et al* 2019). In 2015, the United Nations (UN) (with global support) raised the Sustainable Development Goals (SDGs) aiming to address the maintenance of ecosystem functions and services to both current and future generations (UN 2015). Altogether, the 17 SDGs target to cease poverty and other deprivations while promoting education, equality, food security, and sustainable economic development. Food security remains a great challenge for several countries around the globe as hunger and undernourishment continue to increase (FAO *et al* 2019). In the future, it may become even harder to achieve considering current trends of climatic changes (Schmidhuber & Tubiello 2017) and the current scenario of reduction in the provision of ecosystem services around the globe (Diaz *et al* 2019).

At least 75% of the leading world crops depend, on some degree, on animals for their reproductive success (Klein *et al* 2007); therefore, conservation of pollination services is a major priority for guaranteeing global food security in the long term (Potts *et al* 2016). A robust theoretical foundation has been converted to develop best practices aiming to transcend conventional farming into ecological intensified farming (i.e., replacement of anthropogenic inputs by enhancing Ecosystem Services provision) (Bommarco *et al* 2013, Bommarco *et al* 2018, Garibaldi *et al* 2014, Kleijn *et al* 2019) as a means to ensure biodiversity conservation and food security in sustainable environments. Ecosystem services (ES) are the benefits delivered by nature to guarantee human sustain and well-being (Daily *et al* 1997, Constanza *et al* 1997, Braat & de Groot 2012, but see Diaz *et al* 2015). Over the last 20 years, several ES-based conservation strategies, policies, and programs have been raised to assist sustainable development goals in a changing world (Wood *et al* 2018).

Incorporating the economic contribution of pollination services to the market value of dependent crops is an important device for improving land use planning practices focusing on long-term ES provision and nature conservancy (Breeze *et al* 2016). Monetary valuation of pollination services at global (Gallai *et al* 2009), national (Giannini *et al* 2015a), and local scales (Barfield *et al* 2015, Hipólito *et al* 2019) has been accessed by applying the dependence ratio method. This method evaluates the market value of pollination services, taking into account the dependence ratios of animal pollination for crop production (Gallai & Vaissiere 2009, for dependence ratios, see Klein *et al* 2007 for worldwide crops and Giannini *et al* 2015a for Brazilian crops). This approach enables a more accurate valuation, closer to real-life value of pollination services, and helps predicting the potential production loss in the case of pollinator decline or complete disappearance, and its consecutive impacts on food production and human well-being.

Crop production in Brazil has accounted for more than 5% (US$86 billion) of the country’s gross domestic product (GDP) in 2016, according to the Brazilian Institute of Geography and Statistics (IBGE), this being a high value when compared to high-income countries that have less than 2% (Schmidhuber & Tubiello 2017). About 60% of Brazilian crops are pollinator dependent, which account for one third of the country’s agricultural market value (Giannini *et al* 2015a). In addition, about 60% of the food consumed by Brazilian population is derived from pollinator-dependent crops, representing 21 of the 53 major crops (Novais *et al* 2016). Although this is a high and expressive value, it is not yet final, as many local crops are not included in these studies since they are not present in IBGE’s list, especially those in Brazilian north and northeast regions (Giannini *et al* 2015b). A good example of a local crop only recently (PAM 2016) added to IBGE’s crop list is the now worldwide trade açaí fruit (*Euterpe oleracea* Mart.). Açaí production corresponds to about 30% of non-timber production in Brazil; mainly, its production takes place in floodplain forests (várzeas) of northern Brazil and its dependence ratio on pollinators has been recently classified as great (i.e., about 65% of fruit production is related to animal pollination) (Campbell *et al* 2018). Although açai production is commonly seen as a product of agroforestry and extractivism activities, this perspective is changing given its current market value (Brondizio *et al* 2002), and both floodplain and mainland açai monoculture production systems are rising (Brondizio 2004, Weinstein & Moegenburg 2004, Silva *et al* 2020, Silva *et al* 2019).

Northern Brazil is essentially an Amazonian domain area, a biome that is under high anthropogenic pressures historically associated with land use change (Almeida *et al* 2016, Souza-Filho *et al* 2016, Sonter *et al* 2017). Also, pollinator decline has been forecasted for Amazonian bees, birds, and bats in the future (Costa *et al* 2018, Miranda *et al* 2019, Giannini *et al* 2020) and deforestation can contribute to climate change, potentially increasing land surface temperature up to 1.45°C by 2050 (Prevedello *et al* 2019). The resulting impacts of climate change on ES delivered by biodiversity could be detrimental to human well-being (O’Neill *et al* 2018) and is urgent to anticipate them aiming to help on conservation policy and decision-making processes. Recently, a local study (eastern Amazon, Pará state) estimated the pollination service value provided by a protected area to surrounding crop production to be about half a million dollars (Hipólito *et al* 2019), which support the current need for pollinator conservation strategies to keep up the local economy.

The state of Pará (Eastern Amazon) suffers with the highest deforestation rate in the Brazilian Amazon (Brasil, INPE 2019), being infrastructure, power, mining, pasture, and agriculture among its main drivers. Historically, the state’s economy has been considered to be based on the extraction of natural goods (extractivism activities, i.e., timber, minerals, seeds, and fruits) (Camilotti *et al* 2020, Iorio & Monni 2020) and, although expanding, crop production is considered a marginal aspect of the local economy.

Our objectives are to evaluate the crop pollination services in Pará state (Eastern Amazon, Brazil) and understand the role of agriculture to the state’s economy. We focus on answering two questions: (1) What is the economic value of crop production and pollination service in Pará? (2) Which municipalities are most dependent on pollination services considering their local economies? We aim to highlight regions and municipalities in the state where crop production and pollination services play a main role in the local economy and where public policies on pollination conservation are more urgent to safeguard socioeconomic development and food security.

## Material and Methods

### Study location

The Pará state is the second largest state in Brazil and the 13th largest state in the world. It is located on the eastern portion of the Brazilian Amazon basin and encompasses an area of more than 1.2 million km^2^. The population is estimated to be 8.5 million inhabitants, with a 0.646 HDI (Human Development Index), among the lowest in the country (24 out of 27) (IBGE 2017). The state is divided into 144 municipalities, with a considerable variation in extent (from 103.34 km^2^ in Marituba to 159,533.32 km^2^ in Altamira, the largest Brazilian municipality, being larger than Switzerland) and population (from 3310 in Bannach to 1,485,732 at the state’s capital, Belém) (IBGE 2018). Based on the production structure and spatial interactions, Brazilian municipalities are grouped into microregions (IBGE 1990), which support a better understanding of socio-economic traits at local scale. Therefore, Pará state is divided into 22 microregions that group from 2 to 13 municipalities together.

The state is an Amazon domain area, mainly composed by forest formations, but also presenting natural areas of open vegetation (Pires & Prance 1985). Presently, Pará is located at the eastern portion of the Amazon arc of deforestation (areas of the legal Brazilian Amazon under highest anthropogenic pressures and that present the highest rates of deforestation). Of the 144 municipalities, 17 have the status of Priority Municipalities for conservation actions (Assunção & Rocha 2019), a list created by the Brazilian Ministry of Environment to target the 45 municipalities with higher deforestation rates in the Brazilian Amazon. In 2016, the gross domestic product of Pará state was about US$ 37 billion and about 12% of this value was related to farming production (both livestock and crop production). The remaining value is related to industry, services, and extraction of natural resources (e.g., seeds, timber, and minerals) (IBGE 2018). About one fourth of the state’s working force is employed in agriculture activities (980 thousands out of 3.8 million people, both in crop and in pasture activities), being one sixth the average for the country (IBGE 2018). Pará is one of the biggest markets for tropical fruits in Latin America and has arisen in the national context for its potential for power and natural resources production, being considered the new frontier for capital expansion in Latin America (Iorio & Monni 2020).

### Economic value of crop production

We acquired data on crop production value from the Brazilian Institute of Geography and Statistics (IBGE) for each crop produced in Pará state and for each municipality for the year of 2016 (Electronic Supplementary Material 1). For three municipalities (Belém, Marituba, and Benevides), information on crop production for 2016 was not available; for this, we used data of 2017.

### Pollination service valuation

To estimate the economic market value of pollination services provided by animals to agricultural crops in Pará state (Brazil), the economic production value of each crop was multiplied by the animal pollinator dependency ratio (DR) for crop production, according to the dependence ratio method, proposed by Gallai *et al* (2009). We used DR values following the classifications of Klein *et al* (2007) (international crops), Giannini *et al* (2015a) (Brazilian crops), and Campbell *et al* (2018) (for the local açaí crop), as follows: (i) essential (crop dependence of 90 to 100% of animal pollinators, DR = 0.95); (ii) high (from 40 to 90%, DR = 0.65); (iii) modest (between 10 and 40%, DR = 0.25); and (iv) little (between 0 and 10%, DR = 0.05), according to the classification of Klein *et al* (2007).

In addition to looking at individual crops and individual municipalities, we examined crop production and pollination services accounting for IBGE microregion limits in the state (Electronic Supplementary Material 1). This was done to provide information that can be used in multiple scales by municipality and state governments, stakeholders, and decision-makers into the development of strategies and policies for economic development and pollination conservation. Furthermore, given the high value and importance of açai to local economies, we also calculated the crop production value and pollination service for each municipality without this crop; this was done to demonstrate the impact of this crop to the state’s economy.

### Dependence of municipalities on crop pollination services

For each municipality of Pará State, we determined the annual agricultural crop value and the pollination service value following the same procedure abovementioned. From IBGE, we acquired data on the total gross domestic product (GDP) of Pará and all municipalities in Pará state for the year of 2016 (Electronic Supplementary Material 2). We calculated the percentage of GDP related to the value of pollination service per each municipality in order to estimate a degree of dependence on crop pollination services. It is important to note that the açaí trade, which has high value in the state of Pará, is largely still based on informal markets, and much of its production is not included in the GDP calculation. This is particularly noticeable in the municipality of Igarapé Miri, where açaí production far exceeds total GDP value of the municipality. This will be discussed later (see the Discussion section).

## Results

### Economic value of crop production in Pará

We found 36 crops produced in Pará state (Table 1). Fifteen crops cultivated in Pará present no dependence for animal pollinators, and for one crop (i.e., Brazilian lemon, *Citrus latifolia*), there is no available data regarding its pollinator dependence for fruit production. Among the 15 crops produced in Pará state that we classified as not dependent, two crops were not previously classified by Giannini *et al* (2015a), sorgo, and palm heart, although they were previously included to IBGE’s list. Sorgo is an herbaceous plant of Poaceae family, a predominantly autogamous and hermaphroditic species that does not require animal pollination for seed production (Stephens & Quinby 1934, Muraya *et al* 2011). In Pará, most of the palm heart production comes from açaí palms; although açaí palm fructification has a great dependence on animal pollination, we do not consider palm heart production to be directly related to animal pollination; thus, its production was also classified as not dependent.

**Table 1:** Crops produced in Pará state, their dependence on pollinators and pollination service value.

Crop	Dependence on pollinators	Dependence rate	Crop production value (2016) (US$)	Pollination service value (US$)
Açaí	Great	0.65	977,837,000	635,594,050
Cocoa (almond)	Essential	0.95	197,486,500	187,612,175
Soybean (grain)	Modest	0.25	393,745,250	98,436,313
Watermelon	Essential	0.95	27,441,250	26,069,188
Orange	Modest	0.25	36,856,500	9,214,125
Passion fruit	Essential	0.95	9,339,000	8,872,050
Coco	Modest	0.25	27,222,500	6,805,625
Oil palm (coconut bunch)	Little	0.05	95,619,500	4,780,975
Tomato	Great	0.65	4,535,250	2,947,913
Bean (grain)	Little	0.05	20,863,250	1,043,163
Guava	Great	0.65	1,425,500	926,575
Papaya	Little	0.05	6,508,750	325,438
Cashew nut	Modest	0.25	874,250	218,563
Avocado	Great	0.65	245,750	159,738
Coffee (grain) Total	Modest	0.25	310,250	77,563
Annatto (seed)	Little	0.05	1,338,500	66,925
Guarana (seed)	Great	0.65	56,250	36,563
Tangerine	Little	0.05	351,500	17,575
Melon	Essential	0.95	15,250	14,488
Peanuts (shell)	Little	0.05	49,500	2475
Manioc	No increase	0	483,324,000	0
Black pepper	No increase	0	209,045,500	0
Banana (bunch)	No increase	0	167,798,500	0
Corn (grain)	No increase	0	117,626,250	0
Pineapple	No increase	0	92,205,750	0
Rice (shell)	No increase	0	37,331,000	0
Sugar cane	No increase	0	19,398,000	0
Brazilian Lemon	Unknown	–	17,837,500	–
Palm heart	No increase	0	1,203,250	0
Sorghum (grain)	No increase	0	1,054,000	0
Rubber (coagulated lathe)	No increase	0	855,250	0
Mallow (fiber)	No increase	0	328,750	0
Onion	No increase	0	157,500	0
Mango	No increase	0	113,000	0
Sweet potato	No increase	0	97,000	0
Smoke (leaves)	No increase	0	22,750	0
		Total	2,950,519,500	983,221,475

**Fig 1:**
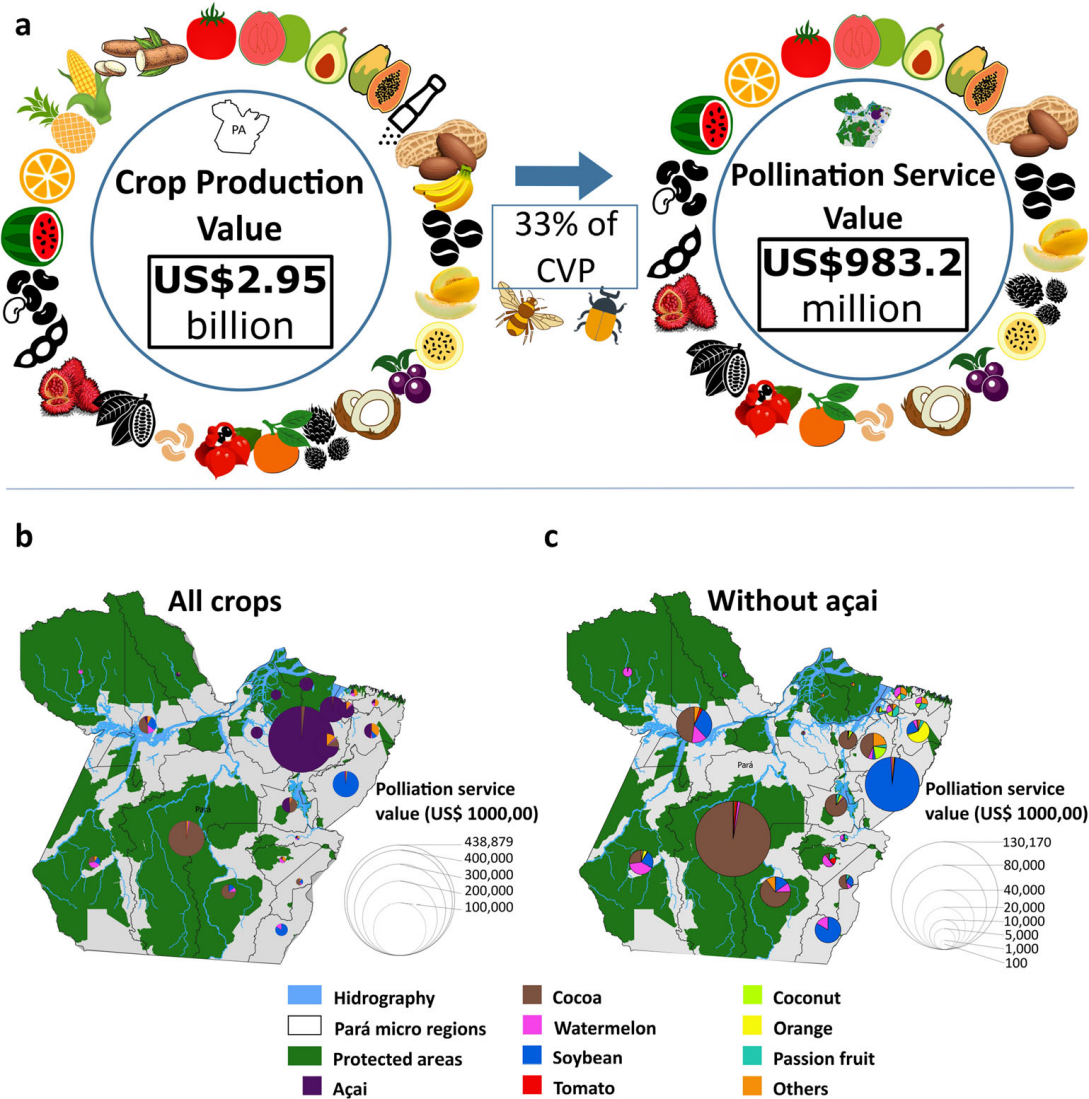
(**a**) The value of crop production and pollination services in Pará state; (**b**, **c**) crops with higher pollination service value for each micro region of Pará state, (**b**) all crops, and (**c**) without açai value.

In 2016, the total crop production value (CPV) for Pará state was of about US$ 2.95 billion (Fig 1A). Five crops with the highest CPV accounted for more than 76% of the state’s CPV (açaí, manioc, soybean, black pepper, and cocoa) (Table 1). From the 10 crops with the highest CPV, four crops depend on animal pollination for fruit set, ranging in dependence from essential (cocoa) to little (oil palm) (Table 1). Açai production corresponds to one third of crop production in Pará (US$ 977 million); therefore, crop production in the state without açai would be reduced to US$1.9 billion (Electronic Supplementary Material 1).

### Economic value of pollination services

We estimated the total pollination service value (PSV) to be US$ 983.2 million (Fig 1A) in 2016 that is 33% of CPV for Pará (Table 1). The crops with highest PSV were açaí palm (US$635.6 million, DR = 0.65), cocoa (US$187.6 million, DR = 0.95), soybean (US$98.4 million, DR = 0.25), and watermelon (US$26.1 million, DR = 0.95) (Fig 1B, C), which accounted for 96% of pollination service value in the state (Table 1). Açai alone accounted for about 64% of PSV, therefore, without açai PSV would be US$347.6 million (Electronic Supplementary Material 2).

Among the 22 microregions in Pará, the Cametá micro region alone presented 45% of the state’s PSV. The micro regions with highest PSV were Cametá (US$400 million), Altamira (US$130 million), Paragominas (US$68 million), Belém (US$65 million), and Tomé-Açu (US$59 million) (Table 2). Açai is the main crop produced in three of the highest PSV microregions (Cametá, Belém and Tomé-Açu), cocoa is the main dependent crop in Altamira micro region, and soybean is the main dependent crop in the Paragominas microregion. Watermelon production is spread throughout the state in all 22 microregions, having the higher values in Paragominas, Itaituba, and Santarem microregions respectively (Fig 1B).

**Table 2:** The twenty-two microregions in Pará, their total crop production value, and pollination service value.

State microregion	Crop production value (2016) (US$)	Pollination service value (2016) (US$)
Cametá	750,213,500	438,878,787
Paragominas	358,140,500	68,100,837
Tomé-Açu	263,353,750	59,193,300
Altamira	200,054,000	130,965,162
Guamá	182,894,000	17,619,400
Santarém	182,351,000	30,947,475
Conceição do Araguaia	158,425,000	17,922,825
Tucuruí	118,008,250	22,995,137
Belém	103,788,000	65,385,875
Bragantina	94,947,750	4,810,475
Castanhal	90,014,750	27,098,812
Itaituba	87,384,750	13,304,412
São Felix do Xingu	71,774,000	21,426,175
Portel	46,079,750	14,659,025
Parauapebas	44,496,250	4,373,212
Óbidos	43,582,500	2,129,262
Arari	41,718,500	20,267,562
Redenção	38,396,000	4,755,012
Marabá	28,876,500	1,899,075
Salgado	23,654,750	5,148,512
Furos de Breves	19,431,750	10,951,112
Almeirim	5,497,000	1,959,237

### Dependence of municipalities on crop pollination services for their local economy

**Fig 2:**
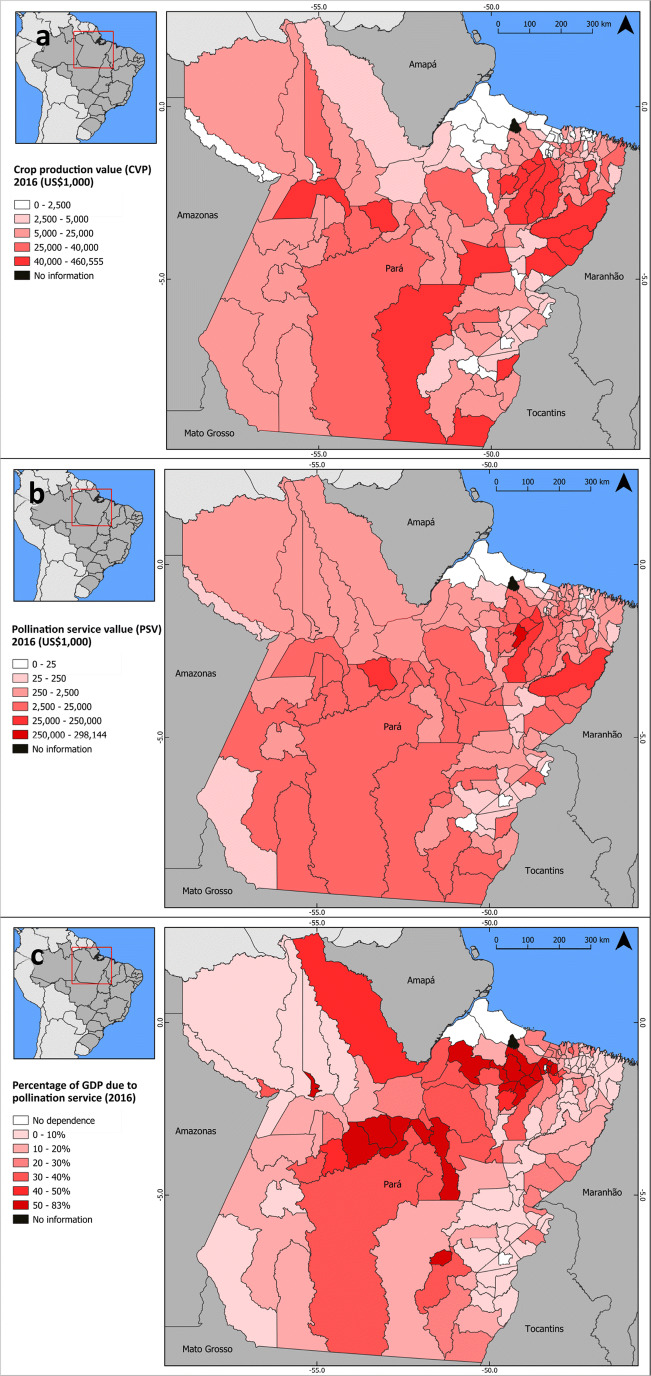
(**a**) Crop production value (CPV); (**b**) pollination service value (PSV) and (**c**) dependence on pollination service (percentage of GDP due to pollination service) of each municipality in Pará.

Crops were produced in 143 municipalities; only one municipality (Santa Cruz do Arari) presented no crop production (Fig 2A; Electronic Supplementary Material 2). The highest CPV can be found on eastern portion of Pará (Fig 2A), while the highest PSV can be found on northeastern areas (specially on Moju, Igarapé-Miri, and Abaetetuba), and in an isolated municipality on the central portion (Medicilândia) (Fig 2B).

The number of crops produced in the municipalities ranged from 1 to 23. CPVs ranged from about US$35 thousand in Soure (lowest CPV) to about US$460 million in Igarapé Miri (highest CPV). The 20 municipalities with the highest CPV accounted for 62% of crop production in the state, and one municipality alone (Igarapé Miri) accounted for 15.6% of CVP in Pará (Electronic Supplementary Material 2). As for PSV, Igarapé Miri and Abaetetuba presented the highest values, respectively, US$ 298 and US$106.8 million, whereas Soure and Palestina do Pará presented the lowest values (US$8.7 and US$1.3 thousand respectively).

Among the 144 municipalities, five do not rely on animal pollination for crop production and 64 have less than 1% of their GDP dependent on pollination services (Electronic Supplementary Material 2). For 62 municipalities, GDP dependence ranged from 1 to 10%, and two municipalities (Medicilândia and Igarapé Miri) have more than 50% of their GDP based on pollination services. In thirteen municipalities (Table 3), one of three crops, açai (7 municipalities), cocoa (4 municipalities), and soy (2 municipalities), accounted for more than 50% of total CPV and four municipalities have more than 97% of their CPV associated with açai alone (Electronic Supplementary Material 2) (Fig 2C).

**Table 3:** The thirteen most dependent municipalities considering the percentage of GDP related to pollination service.

Municipality	Number of crops	GDP (US$)	Total crop production value (2016) (US$)	Main crop (% of total CPV)	Pollination service value (2016) (US$)	GDP % pollination service
Igarapé Miri (PA)	14	91,838,500	460,555,250	Açaí (99%)	298,143,775	324.64^1^
Medicilândia (PA)	18	141,450,000	86,838,500	Cocoa (86%)	72,107,363	50.98
Abaetetuba (PA)	18	312,313,750	168,501,250	Açaí (97%)	106,839,000	34.21
Muaná (PA)	7	58,894,000	21,906,500	Açaí (99%)	14,170,338	24.06
Placas (PA)	15	65,144,500	27,122,000	Cocoa (51%)	13,542,738	20.79
São Sebastião da Boa Vista (PA)	2	44,696,500	13,019,000	Açaí (99%)	8,450,000	18.91
Uruará (PA)	17	125,868,750	30,064,500	Cocoa (65%)	20,167,738	16.02
Brasil Novo (PA)	16	57,163,250	11,527,500	Cocoa (75%)	8,294,363	14.51
Inhangapi (PA)	13	27,833,000	9,536,250	Açaí (54%)	3,761,625	13.51
Moju (PA)	18	217,053,750	74,713,000	Açaí (52%)	28,025,688	12.91
Bujaru (PA)	11	122,488,500	30,928,000	Açaí (73%)	14,905,550	12.17
Dom Eliseu (PA)	17	164,022,000	92,037,500	Soybean (78%)	18,733,275	11.42
Mojuí dos Campos (PA)	20	34,328,250	17,408,250	Soybean (53%)	3,487,988	10.16

^1^This result is due to the informal trade of açaí, which is not included in GDP

## Discussion

Understanding the value of crop production and pollination services in both local and regional scales is vital for conservation planning in order to achieve the global biodiversity and sustainable development targets and food security to human populations in the long term (sustainably) (Wood *et al* 2018, Christmann 2019). In Pará state, twenty (out of 36) crops are dependent on animal pollinators. Pollination service value (PSV) is equivalent to approximately 33% of crop production value (CPV), and the total PSV is approximately equal to US$983 million (year 2016) (Fig 1A). Two crops were highlighted with the highest CPV and PSV, açaí and cocoa. Thirteen municipalities (Table 3) have more than 10% of their GDP associated with pollination services and are, therefore, considered more dependent on crop pollination for their economic stability (Fig 2C). Among them, Igarapé Miri and Abaetetuba (especially due to açaí) and Medicilandia (due to cocoa) are the three municipalities most dependent on pollination services.

A similar percentage of PSV, when considering total CPV, was previously obtained for Brazil (30%; Giannini *et al* 2015a), being soybean and coffee highlighted for Brazil as presenting the highest PSV (Giannini *et al* 2015a). For Pará, four crops presented the highest PSV associated with almost 96% of the total value. Açaí has recently been evaluated as highly dependent on pollinators and involving a complex system of interactions with bees, beetles, and ants; approximately 200 taxa were collected on açaí flowers (Campbell *et al* 2018). Cocoa, whose pollinator dependence is essential, also has a complex pollination system and a recent review discussed the uncertainty about its effective pollinators (Toledo-Hernandez*et al* 2017); thus, further studies are urgently needed. Soybean (the third crop with the highest PSV in Pará) is considered modestly dependent on pollination and is effectively pollinated by honeybees (*Apis mellifera* L.) (Milfont *et al* 2013, Blettler *et al* 2018), an exotic species in Brazil, that is highly generalist and widely distributed. Nevertheless, increased visitation by wild bees can also increase soybean production (Cunningham-Minnick *et al* 2019). However, little is still known about the role of pollination in soybean production in Brazil, since only one variety was studied (soybean cultivar BRS Carnauba; Milfont *et al* 2013), but for Pará this crop represents 10% of total PSV. The fourth crop with highest PSV is watermelon (essential dependence), with studies showing the importance of both stingless bees and honeybees as effective pollinators in Brazil (Bomfim *et al* 2014, Souza & Malerbo-Souza 2005).

Overall, crop production in the state is strongly associated with land use (and deforestation) and water resources (Fig 1B). Soy production is strongly related to old deforestation frontiers (Gasparri *et al* 2013, Nepstad *et al* 2014) and its production in Pará is mainly concentrated on the eastern portion of the state, which coincides with most of the deforested areas in the Amazon deforestation arc. Cocoa production comes mainly from agroforestry system and is concentrated in the south and western portions of the state, where there is great concentration of protected areas. Açai production is concentrated around the state’s capital, Belém, mainly in floodplains, but also in mainland, and constitutes the basis of economy, labor, and food security for traditional and low-income populations in the region (Silva *et al* 2019).

Historically, açai consumption went from local communities to urban centers together with population exodus, achieving national and later international markets as a fashion and healthy food product that represents the support to traditional knowledge, and sustainable food production (Brondizio 2004). However, the industrialization phase of this crop (in the 1990’s and 2000’s) led to severe land use changes by supporting monoculture development in both floodplains and mainland (Weinstein & Moegenburg 2004). Together with land use impacts, the socio-political history in the region produces different returns to local producers, which have low access to infrastructure and economic returns (Brondizio 2004, Silva *et al* 2019). Nevertheless, açai production represents the main source of income for local villages of several municipalities in Pará and one third of total crop production value in the state, presenting a great importance for the state’s economy and food security.

Thirteen municipalities had more than 10% of their GDP associated with PSV (Electronic Supplementary Material 2). Among them stands out Igarapé Miri with high açaí production and informal market, whose values are clearly not incorporated into GDP or local databases. These thirteen municipalities are the more dependent on pollinators and public policies towards the conservation of pollinating insects, as well as ecological intensification farming practices are particularly important for their economic development and population well-being (Kleijn *et al* 2019) (Table 3). Here we have shown how agriculture plays a main role in the local economy of Eastern Amazon municipalities, but still there is a pressing need to better understand the local economy structure, the role of pollination services, and the threats posed by climate and land use changes to human livelihoods. The non-inclusion of açai production to local GDPs provides a glimpse to the lack of local data and knowledge from this region.

Highly dependent municipalities in Pará had more than 50% of CPV associated only to three crops, and four municipalities have more than 97% of their CPV associated with açaí alone (Abaetetuba, Igarapé Miri, Muana, São Sebastião da Boa Vista). The diversity of agricultural crops was previously related to the concept of resilience (Gbetibouo *et al* 2010), because high diversity implies a greater chance of assimilating possible impacts or reductions in the production of one or a few crops. Also, crop diversity would support more agricultural jobs, grounding local livelihoods and socio-economic development (Garibaldi & Pérez-Mendez 2019). Thus, it can be suggested that municipalities whose production depends solely on a single crop discuss their current socio-economic plans, aiming to enhance crop diversification.

Public policies for pollinator conservation have already been suggested, being particularly important for the conservation of natural areas near or within crops (Garibaldi *et al* 2014). This is particularly important for açaí, a crop mainly pollinated by small stingless bees (such as small *Trigona*-like bees, Campbell *et al* 2018), with short flight ranges and more commonly found on well-preserved habitats, due to their nesting requirements (Borges *et al* 2020); in fact, crops near forested areas presented higher fruit production (Campbell *et al* 2018). In this sense, conservation of legal reserves and maintenance of forest patches within rural areas is a particularly important mechanism in Brazil for the conservation and sustainable use of biodiversity (Garibaldi *et al* 2011, Metzger *et al* 2019), which should be encouraged and regulated, especially in the Amazon biome states (Freitas *et al* 2015, Christmann 2019, Metzger *et al* 2019, Nunes *et al* 2019).

Protected areas are also important to safeguard pollinator diversity and deliver crop pollination services (Hipólito *et al* 2019). In fact, agricultural production is key to ensure sustainable development (DeClerck *et al* 2016, Garibaldi & Pérez-Mendez 2019) and is associated with all the 17 Sustainable Development Goals (SDGs), integrating the three dimensions of sustainable development—economic growth, social inclusion, and environmental protection (FAO 2018). In addition, tropical forest conservation has been increasingly associated with socio-economic development through the provision of various ecosystem services (Constanza *et al* 1997, 2014), and a better understanding of non-listed local crops as well as their effective pollinators is required for the development of local strategies. The value of standing forest exceeds other land uses, and deforestation can result in high social (Franklin & Pindyck 2018) and economic costs (Hipólito *et al* 2019). Integrating long used local crops to socio-economic systems seems to be a fundamental tool for developing sustainable development in forest ecosystems. In a rapidly changing world, anticipating the impact of climate change is also indispensable and scenarios for pollinators in the state of Pará have been forecasted, suggesting that crop pollinator bees will potentially be highly affected by climate changes by 2050 (Giannini *et al* 2020).

Future work should address the knowledge gap about the identification of crop pollinators for Amazonian agricultural crops, as still little is known about the species that provide this service. Additionally, many crops of regional interest consumed by local fisherman communities (Ribeirinhos) have not yet been studied; the pollination system of their farming activities is little known and its importance for family farming in Amazonian traditional communities has not been assessed.

## Electronic Supplementary Material

ESM 1 Crop production value for each crop and for each municipality in Pará state for the year 2016 (XLSX 61 kb)

ESM 2 Number of crops, gross domestic product value, total crop production value, total pollination service value and values without açai for each municipality and for each micro region in Pará state for the year 2016 (XLSX 36 kb)
